# Incubation temperature and physiological aging in the zebra finch

**DOI:** 10.1371/journal.pone.0260037

**Published:** 2021-11-29

**Authors:** Henrik H. Berntsen, Claus Bech

**Affiliations:** 1 Department of Biology, Norwegian University of Science and Technology (NTNU), Trondheim, Norway; 2 Norwegian Institute for Nature Research, Trondheim, Norway; University of Missouri Columbia, UNITED STATES

## Abstract

In birds, incubation temperature has received increased attention as an important source of phenotypic variability in offspring. A lower than optimal incubation temperature may negatively affect aspects of nestling physiology, such as body growth and energy metabolism. However, the long-term effects of sub-optimal incubation temperature on morphology and physiology are not well understood. In a previous study, we showed that zebra finches from eggs incubated at a low temperature (35.9°C) for 2/3 of the total incubation time suffered a lower post-fledging survival compared to individuals that had been incubated at higher temperatures (37.0 and 37.9°C). In the present study, we investigated whether these variations in incubation temperature could cause permanent long-lasting differences in body mass, body size, or basal metabolic rate. Furthermore, we tested whether the observed differences in survival between treatment groups would be reflected in the rate of physiological deterioration, assessed through oxidative damage and decreased metabolic rate with age (i.e. ‘metabolic aging’). Incubation temperature did not significantly affect embryonic or nestling body growth and did not influence final adult body mass or body size. Nor was there any long-term effect on basal metabolic rate. Birds from eggs incubated at the lowest temperature experienced an accumulation of oxidative damage with age, although this was not accompanied by an accelerated rate of metabolic aging. The present results suggest that the low survival in these birds was possibly mediated by increased oxidative stress, but independent of body growth and the basal metabolic rate.

## Introduction

Environmental conditions experienced early in life play a key role in determining individual quality [1]. Hence, by providing certain developmental environment, parents may help shape the phenotype of their offspring via non-genetic contributions [2]. Because the growth of avian embryos are particularly sensitive to temperature [3], incubation temperature has received increased attention as an important parental effect in birds [4]. Incubation is, in addition, an energetically costly behavior [5, 6] and is influenced by both physical environmental conditions as well as the physiological state of the parents [7–11]. Consequently, when energetically challenged, parents may need to trade off maintenance of incubation temperature for self-maintenance [12, 13] with the resulting change in incubation temperature having negative implications for embryonic growth.

Experimental studies using both precocial and altricial species have shown that even slight deviations from the optimal incubation temperature (although still within the natural range) negatively affect offspring development (reviewed in [4]). In addition to taking longer to develop, embryos incubated at low temperatures also exhibit a reduced ability to convert yolk into tissue [14, 15], have higher metabolic rates [16] and expend more energy during incubation [17]. As a result, chicks from eggs incubated under low temperatures often hatch with fewer energy reserves, a lower body mass and are in poorer condition [14, 18, but see 19, 20]. Furthermore, this reduced embryonic growth efficiency also seems to carry over into the nestling stage. Nestlings from eggs incubated under low temperatures exhibit higher metabolic rates [20], reduced growth rates [20, 21] and lower body condition [19, 22, 23, but see 24]. Clearly, incubation temperature has a strong influence on the early (pre- and early post-natal) physiological quality of offspring and the ability of the parents to maintain an optimal incubation temperature may consequently play a critical role in determining the life-history trajectory of their offspring [25]. Nevertheless, if developmental conditions are to influence individual life-long performance and fitness, the phenotypical effects that are evident during early life must necessarily carry over into the adult life [1]. However, whether incubation temperature can cause such permanent effects on individual phenotypes beyond the nestling stage is still not well known.

Although early growth conditions can have immediate effects on parameters such as body mass or body size, other effects on physiological function may not become evident until later in adult life. Aging or senescence constitutes a progressive loss of physiological function with advancing age [26] and oxidative stress plays an important role in the aging process. Oxidative stress constitutes the damage to biomolecules such as proteins, lipids and DNA that occurs from an imbalance between the production of reactive oxygen species (ROS) from aerobic metabolism and the capacity of antioxidant defense and repair systems. The accumulation of oxidative damage with age is believed to be one of the key mechanisms underlying cellular and organismal senescence [27, 28]. In both mammals and birds, the quality of the developmental environment has been shown to be particularly important in determining individual variation in aging patterns [25, 29]. When faced with sub-optimal developmental conditions, an individual may trade-off long-term performance (i.e., self-maintenance and survival) for early life growth and maturation [25, 30]. As oxidative stress is suggested to mediate such life-history trade-offs [27, 31], a reduced investment in somatic self-maintenance processes (e.g., regulation of oxidative stress) may consequently manifest through an accelerated loss of physiological function with advancing age.

The zebra finch (*Taeniopygia guttata*) is a small passerine bird with a lifespan of approximately 5.5 years [32]. In a previous study [33], we showed that a slight variation in incubation temperature affected survival in captive zebra finches. We experimentally manipulated incubation temperature by artificially incubating eggs at three temperatures within the natural range and found that individuals incubated at the lowest temperature had a significantly reduced long-term survival compared to those incubated at the highest temperature. In the present study, we wanted to further investigate the effects that incubation temperature had on long-term physiological performance using the same birds. To this end, we monitored physiological parameters reflecting aspects of individual quality such as body mass and body size, metabolic rate, and plasma oxidative status over a period of two and a half years from hatching. Our aims were two-fold. Firstly, we wanted to test whether the variation in incubation temperature could influence both pre- and post-natal growth and if any effects on body mass or body size would be permanent. Specifically, we hypothesized that a low incubation temperature would be associated with reduced growth rates resulting in a smaller adult body mass and size. Secondly, we investigated whether incubation temperature could influence the investment in somatic self-maintenance processes such as metabolic rate and oxidative status. Because mitochondria are regarded as the primary producers of ROS (but see Zhang and Wong [34]), the mitochondria are inevitably a major site of oxidative stress. Consequently, as oxidative damage accumulates mitochondrial function deteriorates, resulting in a change in metabolism with age [35, 36]. Basal metabolic rate (BMR) is known to decline with age in the zebra finch [37, 38]. Because the rate of metabolism is determined by mitochondrial function, rapid accumulation of oxidative damage in mitochondria should result in a rapid loss of mitochondrial function and consequently an accelerated rate of decline in BMR with age. Given this relationship, we hypothesized that a low incubation temperature would negatively influence the investment in self-maintenance processes, resulting in high levels of oxidative stress and consequently an accelerated rate of decline in BMR (i.e. ‘metabolic aging’).

## Materials and methods

### Experimental setup

For a detailed description of the experimental set-up, see Berntsen and Bech [33]. In short, male, and female zebra finches were bred in three large walk-in aviaries, all equipped with nest boxes. Nest-boxes were checked for breeding activity every day and newly laid eggs were weighed on a digital scale (Sartorius, AG Göttinger, Germany) to the nearest 0.001 g and individually marked with a pencil. On the second day after clutch completion, we removed all eggs from the nest-boxes and artificially incubated these for eight days (corresponding to two-thirds of the total incubation time) in commercial incubators (America AS, Thisted, Denmark) at mean temperatures of 35.9, 37.0 and 37.9 ± 0.2°C and at a relative humidity of 70%. These temperatures fall within the natural range of incubation temperatures in the zebra finch [32] and represents low, intermediate and control temperatures respectively [33]. At removal from the nest boxes the eggs (i.e., full clutches) were substituted by dummy eggs made from plaster, matching the original clutch in both eggs size (as close as possible) and number. Throughout the experiment, all nests were checked daily to make sure that incubation was not affected by the experimental treatment. After eight days of external incubation, eggs were returned to their nest of origin and naturally incubated by their parents until hatching. Clutches showing signs of non-normal incubation (e.g., egg neglect) at this point were excluded from the study. Incubation time was noted for each individual egg and was measured as the time from initiation of full incubation (eggs warm to the touch, corresponding to the time of clutch completion) until hatching. All chicks hatched in the nest-boxes.

The experimental treatment was administered at the clutch level and where a female laid more than one clutch the treatment was altered between clutches. In this way, we were able to have multiple levels of the experimental treatment represented within a single female (mother). However not all females laid multiple clutches. Hence, females laying three clutches had each clutch incubated under different experimental temperatures. For those females that laid two clutches the experimental treatment was altered between the clutches and also between females in order to produce all possible (three) combinations of treatments, i.e. 35.9–37.0°C, 37.9–35.9°C and 37.0–37.9°C respectively. From the 29 females that reproduced, four females contributed three clutches, 12 females contributed two clutches and 13 females contributed one clutch. See S1 Table for a full overview of the distribution of chicks and associated females (mothers) between treatment groups. Clutch size (both number of eggs and egg mass) and brood size (number of birds in nest) did not differ between treatments (linear mixed model, both P > 0.38) nor were there any significant differences in body mass or body condition (body mass vs. tarsus length) of mothers between treatments or among clutches (linear mixed models, all P > 0.1).

In the breeding colonies all birds received a diet consisting of mixed seeds (Life Care; protein content: 10.8% of wet mass; water content 11.7%) and a commercial protein supplement (Eggfood Witte Molen; protein content: 11.3% of wet mass; water content 9.8%), with an additional mineral supplement (Nekton-S, Nekton Germany) given once a week. During the entire breeding period, we also provided the birds with nest materials such as paper and straw. At the age of 45 days all chicks (n = 85) were removed from their respective breeding aviaries and placed in sex specific holding aviaries. In the holding aviaries, all chicks received a mixed seed diet and the additional mineral supplement. All birds had free access to food and drinking water. In both the breeding colonies and the holding colonies room temperature and humidity was held constant at 23°C and 40% respectively, and the birds received a 12h:12h light-dark regime, with lights on at 08:00. All birds hatched naturally and were followed for close to three years. During this period only naturally occurring mortality was observed. The Norwegian Animal Research Authority (permit number S-0028/01) approved housing and the experimental conditions for the birds.

### Growth parameters

Upon hatching, chicks were individually marked using a non-toxic felt-tip marker and were remarked whenever necessary. At the age of 15–17 days, all birds were banded with a metal ring. We measured body mass of every chick to the nearest 0.01g using a digital balance (Sartorius, AG Göttinger, Germany) on a daily basis from the day of hatching (day 0) until 20 days of age and again at the age of 45 (i.e., at the end of the growth period), 145 and 975 days as sexually mature adults. The increase in body mass from hatching to day 20 was modelled by the three-parameter logistic growth equation derived from [39]; M_x_ = A / (1 + e^-k(x-I)^), where M_x_ is body mass (g) at age X (measured in days), A is the asymptotic body mass (g), k is the growth rate constant (day^-1^) and I is the inflection point (in days) of the growth curve. Values were derived for each individual chick (n = 98) by fitting logistic growth curves in a non-linear regression model in Sigma plot 11.0 (Systat Software Inc., Berkshire, UK).

As a measure of structural size, we also monitored the growth of tarsus length. Tarsus length was measured at day 10, 20, 45, 145 and 975 using a digital slide caliper (Mitutoyo, accuracy 0.01 mm). We also calculated a body condition index (BCI) by taking the (standardized) residuals from a linear regression between log-body mass (dependent variable) and log-tarsus length. All biometric measurements were taken by the same person (HHB), between 12:00 and 14:00.

### Metabolic measurements

We performed metabolic measurements of the individual birds at four different ages: day 15 (n = 88), day 45 (n = 84), day 145 (n = 61) and day 975 (n = 34). Metabolic rate was measured indirectly as rates of oxygen consumption (VO_2_) using an open flow-through respirometry system (see also [40] for details). Outside air was dried using Silica gel (VWR®) before being pumped through four metabolic chambers. The metabolic chambers were made from 1.1 L metallic boxes, which were painted black on the inside and equipped with a perch where the birds could rest. Airflow through the chambers was controlled using flowmeters (Bronkhorst) and held constant at 400 mL of air per minute. A two-channel oxygen analyzer (Servomex, 5400 series) measured the oxygen concentration of the effluent air, which was dried using Drierite^TM^ (Sigma-Aldrich) before entering the oxygen analyzer. An automated valve system switched between the four metabolic chambers so that two chambers were measured simultaneously for 26 minutes every hour. The oxygen analyzer was calibrated using outside air (set to 20.95% oxygen) before every new measurement and pure nitrogen was used for zero calibration. All voltage outputs from the analyzer and mass flow meters were recorded at 30-second intervals and stored on a Grant Squirrel data logger (1200 series).

Metabolic measurements were conducted under thermoneutral (35°C) conditions [41] and all measurements were done between 20:00 and 08:00. Hence, measurement of oxygen consumption was obtained during the night and the lowest 10-min average value was taken to represent the individual metabolic rate. Rates of oxygen consumption were calculated using the formulas given by [42]. Since the lowest values of VO_2_ were obtained early in the morning (between 04:00 and 07:00) the birds were assumed to be post-absorptive; and a respiratory exchange ratio of 0.71 was consequently used. Body mass of the individual birds was measured before and after metabolic measurements and a linear reduction in body mass during the night was assumed for obtaining the body mass associated with the lowest nightly VO_2_. Because the metabolic rates at day 15 are measured during a period of intense growth, these values are taken to represent resting metabolic rate (RMR), while the values at day 45, 145 and 975 represent basal metabolic rate. Values of RMR/BMR are expressed as residuals from a log-log regression between body mass and whole body RMR/BMR for all ages combined (values of whole body BMR are presented in S2 Table).

### Oxidative status

We assessed changes in individual oxidative status, from fledging to achievement of sexual maturity, by measuring the concentration of hydroperoxides in plasma at the age of 45 and 145 days. Blood was sampled from each bird in connection with the metabolic measurements at these two ages when the birds were removed from the metabolic chambers in the morning (i.e., at 08:00). Plasma was separated by centrifugation and stored on -80°C until the time of analysis.

Since avian blood cells, in contrast to mammalian blood cells, contain mitochondria, blood samples are perfect ways to obtain non-invasively measures of Reactive oxygen species (ROS) [43]. These are by-products of aerobic metabolic processes, are extremely reactive molecules that cause damage to lipids, proteins and nucleotides. These reactions generate intermediate compounds (by-products) such as reactive oxygen metabolites (ROMs, primarily hydroperoxides; ROOH) that maintain oxidizing properties, and which can further contribute to the oxidation cascade. ROMs or hydroperoxides therefore function as a proxy of the oxidative damage that has occurred in the organism and also the potential damage that can be caused by these products [44]. The concentration of hydroperoxides (hereafter addressed as ROMs) in plasma was measured following the protocols of [44, 45]. We added 5 μL of plasma to 200 μL of 0.1 M acetic acid/sodium acetate buffer (pH 4.8) before finally adding 5 μL of a 0.37M N,N-diethyl-p-phenylenediamine (Fluka analytical) chromogen solution. In the acidic pH of the acetate buffer, iron (Fe^2+^ and Fe^3+^) is released from plasma proteins. Iron catalyzes the cleavage of hydroperoxides, which generates highly reactive pro-oxidants: alkoxyl (R-O*) and alkylperoxyl (R-OO*) radicals. These compounds in turn react with an amine group in the chromogen producing a complex that is pink in color. The intensity of the color is hence proportional to the concentration of the radicals and can be determined spectrophotometrically.

The absorbance was read with a microplate reader (Synergy HT, Bio-Tek) at 495 nm immediately after adding the chromogen solution and again after 75 min of incubation at 37°C. Samples were run in duplicates on the same plate and each microplate included its own control and standard curve of serial dilutions of H_2_O_2_ (1–0.05 mM). Both the pooled sample (R = 0.81) and intraplate repeatability (range: 0.71 to 0.92, all P < 0.001 [46]) was high and a mean value was used in the analysis. Values are expressed as mmol L^-1^ (mM) H_2_O_2_ equivalents.

### Statistical analyses

All statistical analyses were done using linear mixed effects models (lme4 package [47]) in R, version 3.2.2 for Windows [48]. To account for the dependency of data all models (unless otherwise stated) included brood identity nested within mother identity as a random effect. Mother identity was the observed mother incubating and raising chicks and brood identity was entered in the model with a unique identity for each brood. Included in the statistical analyses are only chicks that survived until and beyond 20 days of age and from which we obtained the relevant measurements. Therefore, sample sizes may vary due to missing data, either because of known measurement errors (removed values) or because measurements are lacking. S2–S4 Tables gives a full overview of sample sizes of all measured variables as well as group means ±SE. All data used in this manuscript is available at https/doi.org/10.5061/dryad.mgqnk990k [49].

#### Pre-natal and post-natal growth

The effect of incubation temperature on incubation time was analyzed with treatment, sex, and their interaction as fixed effects. Clutch size and egg mass were considered as covariates, but were dropped from the final analysis, as both terms were not significant (P > 0.20). Hatchling body mass was analyzed with treatment, sex and their interaction as fixed effects and egg mass as a covariate.

Variation in nestling growth parameters (i.e., growth rate, inflection point and asymptotic body mass) were analyzed in identical models with treatment, sex and their interaction as fixed effects. Brood size (i.e., the number of chicks in the nest) was initially included as a covariate but had no effect on either of the three growth parameters (all P > 0.5) and was therefore not included in the final analysis. In addition to the logistic growth parameters, we also analyzed variation in body mass, tarsus length and body condition index with time (day 10, 20, 45, 145 and 975) as the repeated variable and treatment, sex and the interaction between treatment and time and sex and time as fixed effects.

#### Metabolic rate

In the same way as done for body mass and BCI, variation in metabolic rate was analyzed with time (day 15, 45, 145 and 975) as the repeated variable and treatment, sex and the interaction between treatment and time and sex and time as fixed effects. Metabolic chamber was initially included as a random term, but only explained a small part of the variation in BMR (<0.1%) and was therefore excluded from the final analysis. In addition, we also investigated the effect of incubation temperature on the rate of metabolic aging (i.e., the effect of age on BMR) from day 45 until day 975. This was done using a linear mixed effect model fitted with bird identity as an additional random effect. The full model contained treatment, sex, age (in days) and the interaction between sex and age and treatment and age as explanatory variables. Since we excluded the measurements at day 15 from this analysis values of BMR were re-calculated (i.e., corrected for body mass) in the same way as explained above, but excluding the measurements at day 15.

#### Oxidative status

Plasma oxidative status (i.e., the concentration of ROMs) at day 45 and day 145 was analyzed using identical models including treatment, sex and the interaction between treatment and sex. Values for concentration of ROMs were log10-transformed to meet assumptions of normality.

Model simplifications were carried out with the stepwise removal of non-significant (P > 0.05) fixed effects terms based on likelihood ratio. Post-hoc analyses were conducted using LSD tests. Parameter estimates were obtained by re-fitting final models with REML. Values are presented as means ± SE and are, unless otherwise specified, presented as raw values.

## Results

### Embryonic growth

Incubation temperature significantly affected incubation time (P = 0.003, Tables 1 and 2). Eggs incubated at 35.9°C took on average 1.34 and 0.94 days longer to hatch compared to eggs incubated at 37.9°C and 37.0°C, respectively (post-hoc both P < 0.03). The difference in incubation time of 0.34 days between the 37.0°C and 37.9°C treatment groups was not significant (post-hoc P = 0.21, parameter estimates: 35.9°C: 14.63 ± 0.28 days, 37.0°C: 13.78 ± 0.30 days, 37.9°C: 13.38 ± 0.29 days). No differences were found between the sexes (see full model details in S2 Table).

**Table 1:** Embryonic (incubation time and hatchling weight) and nestling growth parameters for birds incubated at different temperatures.

	Incubation temperature		
Variable	35.9°C (N = 35)	37.0°C (N = 30)	37.9°C (N = 33)
Incubation time (days)	14.37 ± 0.21	13.43 ± 0.18	13.03 ± 0.29
Hatchling mass (g)	0.973 ± 0.032	0.943 ± 0.031	0.985 ± 0.028
Growth rate constant (K, day^-1^)	0.395 ± 0.013	0.412 ± 0.012	0.428 ± 0.010
Asymptotic body mass (A, g)	13.14 ± 0.27	13.41 ± 0.30	13.97 ± 0.32
Inflection point (I, days)	6.92 ± 0.26	6.96 ± 0.23	6.67 ± 0.17

See S1 Table for statistics.

**Table 2:** Results of linear mixed effects models on incubation time, hatchling body mass and growth parameters, growth rate constant (K), asymptotic body mass (A) and inflection point (I) calculated from a logistic growth model.

Variable	Estimate ± SE	χ^2^	df	p
Embryonic growth				
Incubation time				
** Treatment**	**14.63 ± 0.28**	**11.51**	**2**	**0.003**
Sex	NS	1.04	1	0.31
Treatment*sex	NS	3.58	2	0.17
Hatchling mass				
Treatment	NS	1.52	2	0.46
Sex	NS	0.19	1	0.66
** Egg mass**	**0.379 ± 0.116**	**11.43**	**1**	**<0.0001**
Treatment*sex	NS	3.15	2	0.21
Nestling growth parameters				
Growth rate constant (K)				
Treatment	NS	3.23	2	0.19
Sex	NS	0.06	1	0.81
Treatment*sex	NS	2.53	2	0.28
Asymptotic body mass (A)				
Treatment	NS	1.03	2	0.60
Sex	NS	3.51	1	0.06
Treatment*sex	NS	4.33	2	0.12
Inflection point (I)				
Treatment	NS	1.32	2	0.51
Sex	NS	0.47	1	0.49
Treatment*sex	NS	2.30	2	0.31

Significance (p) based on likelihood ratio tests and parameter estimates are given for variables retained in the final model. Estimates are given for Treatment = 35.9°C. Significant terms are highlighted in bold. See Methods and Results for further details.

Egg mass was a significant predictor of hatchling mass (P < 0.0001). However, hatchling mass was not affected by incubation temperature (P = 0.46, Table 2) or by sex (P = 0.66).

### Nestling growth, body mass and body condition

There were no significant differences in either growth rate constant (k), asymptotic body mass (A) or inflection point (I) between treatment groups (all P > 0.19). Hence, the experimental treatment did not affect the growth pattern in body mass from hatching until day 20 (Tables 1 and 2). No differences were found between the sexes for growth rate constant and inflection point (both P > 0.49) and females tended to reach a higher asymptotic body mass than males (Males: 13.26 ± 0.23 (n = 54), Females: 13.79 ± 0.27 (n = 44), P = 0.06). See Table 2 for full model details.

**Fig 1:**
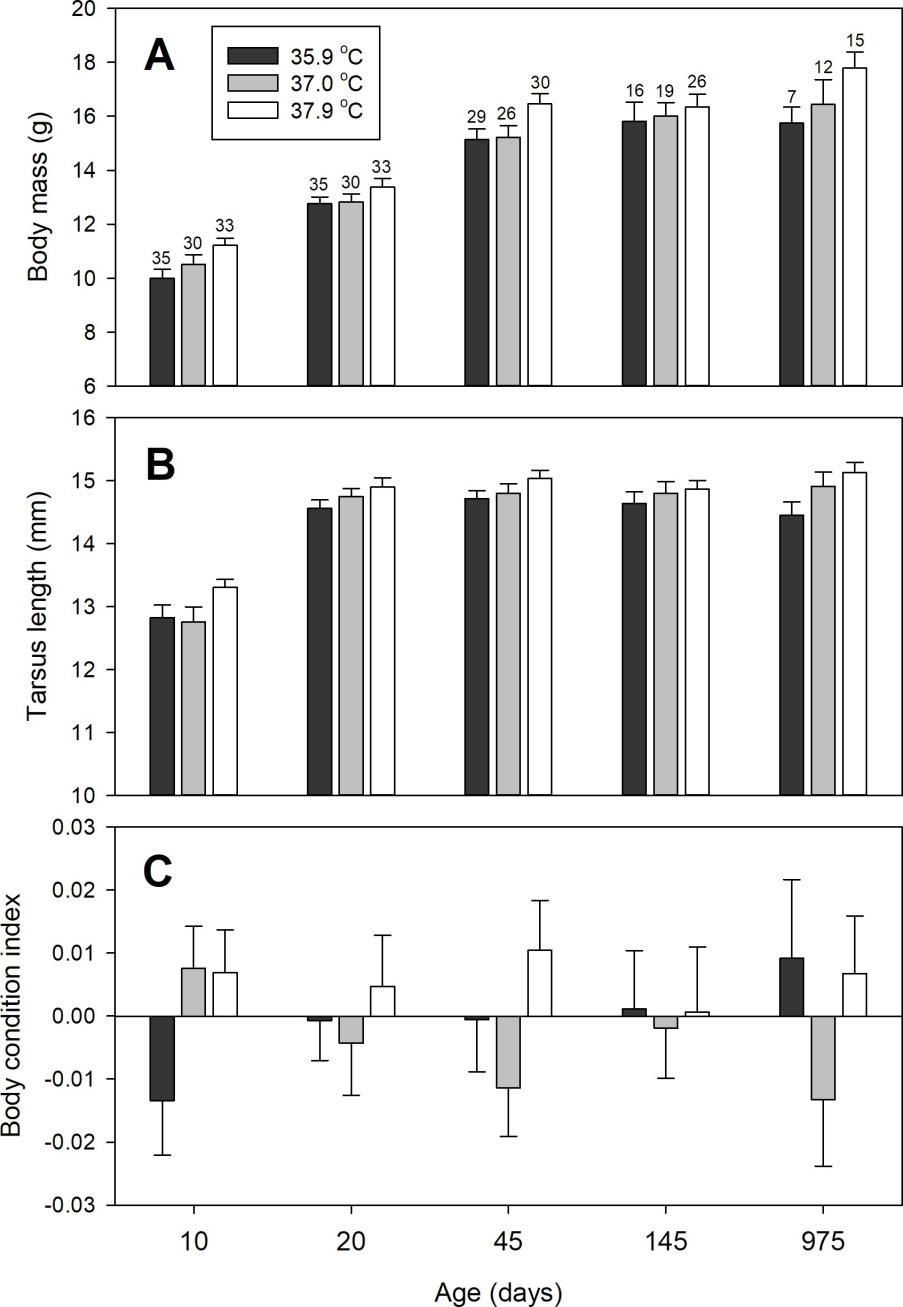
Body mass and biometry changes with age. Body mass (A), tarsus length (B) and body condition index (BCI; C) at different ages for birds incubated at 35.9°C (black), 37.0°C (grey) and 37.9°C (white). Shown are means ± SE. Sample sizes are given above the error bars.

Although treatment means did vary in the predicted direction (Fig 1A) the experimental treatment did not significantly affect body mass during the growth period (day 10 and 20) or afterwards (day 45, 145 and 975) (treatment*time: P = 0.58). Hence, the variation in incubation temperature did not permanently influence body mass. No differences were found between the sexes (full model details in Table 3). Tarsus length was overall also shortest in birds from the low temperature treatment (Fig 1B), however this difference in tarsus length was not statistically significant (treatment*time: P = 0.71). Males and females did not differ in growth of tarsus length (full model details in Table 3). For body condition (BCI) the interaction term between treatment at time was close to significant (treatment*time: P = 0.07). This trend was primarily a consequence of chicks from eggs incubated at the lowest temperature exhibiting a considerably lower body condition at day 10 compared to chicks from the other two treatment groups (Fig 1C). Hence, suggesting a differential growth rates for body mass and tarsus length between treatment groups from hatching up until this age. Full model details are given in Table 3.

**Table 3:** Results of linear mixed effects models on body mass, tarsus length, body conditions index and metabolic rate.

Variable	Estimate ± SE	χ^2^	Df	p
Body mass				
Treatment	NS	1.99	2	0.28
Sex	NS	0.83	1	0.36
** Time (day 10, 20, 45, 145, 975)**	**10.83 ± 0.37**	**432.33**	**4**	**<0.001**
Treatment * time	NS	6.29	8	0.58
Sex * time	NS	2.59	4	0.61
Tarsus length				
Treatment	NS	3.43	2	0.18
Sex	NS	0.81	1	0.37
** Time (day 10, 20, 45, 145, 975)**	**13.03 ± 0.13**	**341.95**	**4**	**<0.001**
Treatment * time	NS	5.50	8	0.71
Sex * time	NS	0.46	4	0.93
Body condition index				
Treatment	NS	1.44	2	0.49
Sex	NS	0.31	1	0.58
Time (day 10, 20, 45, 145, 975)	NS	0.30	4	0.99
Treatment * time	NS	14.70	8	0.07
Sex * time	NS	6.18	4	0.18
Metabolic rate				
Treatment	-	NA	NA	NA
Sex	NS	0.10	1	0.75
Time (day 15, 45, 145, 975)	-	NA	NA	NA
Treatment * time	**3.77e-02 ± 1.06e-02**	**16.26**	**6**	**0.01**
Sex * time	NS	5.10	3	0.17
Metabolic ageing				
Treatment	NS	0.20	2	0.91
Sex	NS	0.06	1	0.81
** Age**	**-9.89e-05 ± 7.58e-06**	**107.4**	**1**	**<0.001**
Treatment*age	NS	1.58	2	0.45
Sex*age	NS	0.70	1	0.40

NA refers to the lack of test statistics for main effects included in the interaction. Significance (p) is based on likelihood ratio tests and parameter estimates are given for variables retained in the final model. Estimates are given for Treatment = 35.9°C and Time = day 10. Significant terms highlighted in bold. See Methods and Results for further details.

### Metabolic rate

**Fig 2:**
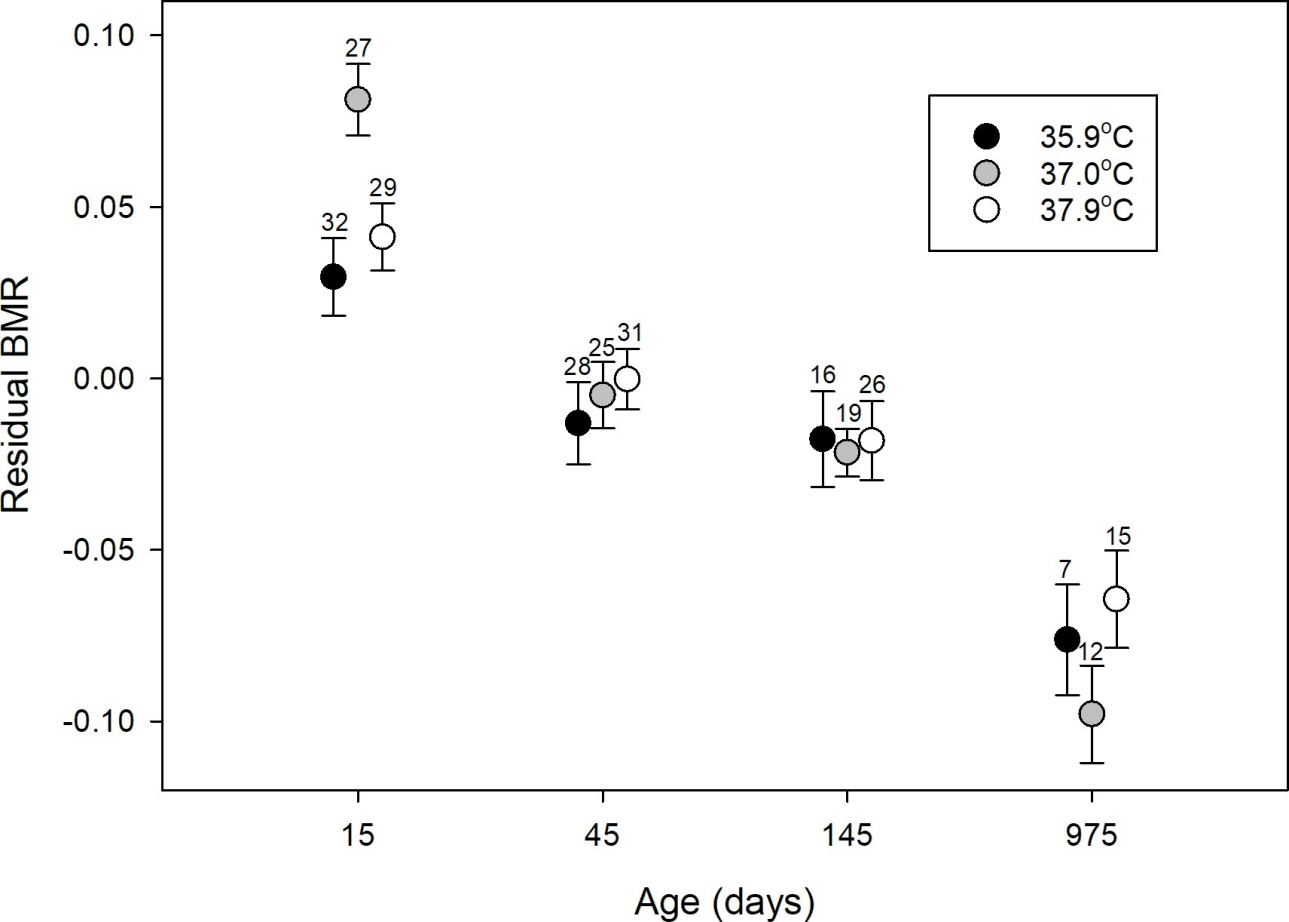
BMR decreases with age. BMR at four different ages for birds, in which eggs were incubated at 35.9°C (black), 37.0°C (grey) and 37.9°C (white). Shown are mean residual values ± SE. Sample sizes are given above the error bars. Metabolic rates are expressed as residuals from a log-log regression between body mass and total RMR/BMR for all ages combined. See S2 Table for actual values of whole body BMR.

A significant variation in metabolic rate was explained by the interaction between incubation temperature and time (treatment*time: P = 0.01, Table 3). At day 15, individuals from eggs incubated at 37.0°C exhibited significantly higher rates of oxygen consumption (i.e., RMR) than individuals from the other two treatment groups (both P < 0.001, Fig 2), while RMR in the lowest and the highest temperature treatments did not differ significantly (P = 0.77, parameter estimates of residuals: 35.9°C: 0.038 ± 0.010, 37.0°C: 0.086 ± 0.012, 37.9°C: 0.034 ± 0.011). This difference in metabolic rate was not, however, long lasting and BMR did not differ between treatment groups at any of the following ages (all P > 0.21, Fig 2). None of the other explanatory variables was found significant. See Table 3 for full model details.

There was a significant decline in metabolic rate with age (P < 0.001). The experimental treatment, however, had no effect on the rate of metabolic decline with age (treatment*age: P = 0.45), which was also independent of sex (sex*age: P = 0.40; Table 3).

### Plasma oxidative status

**Fig 3:**
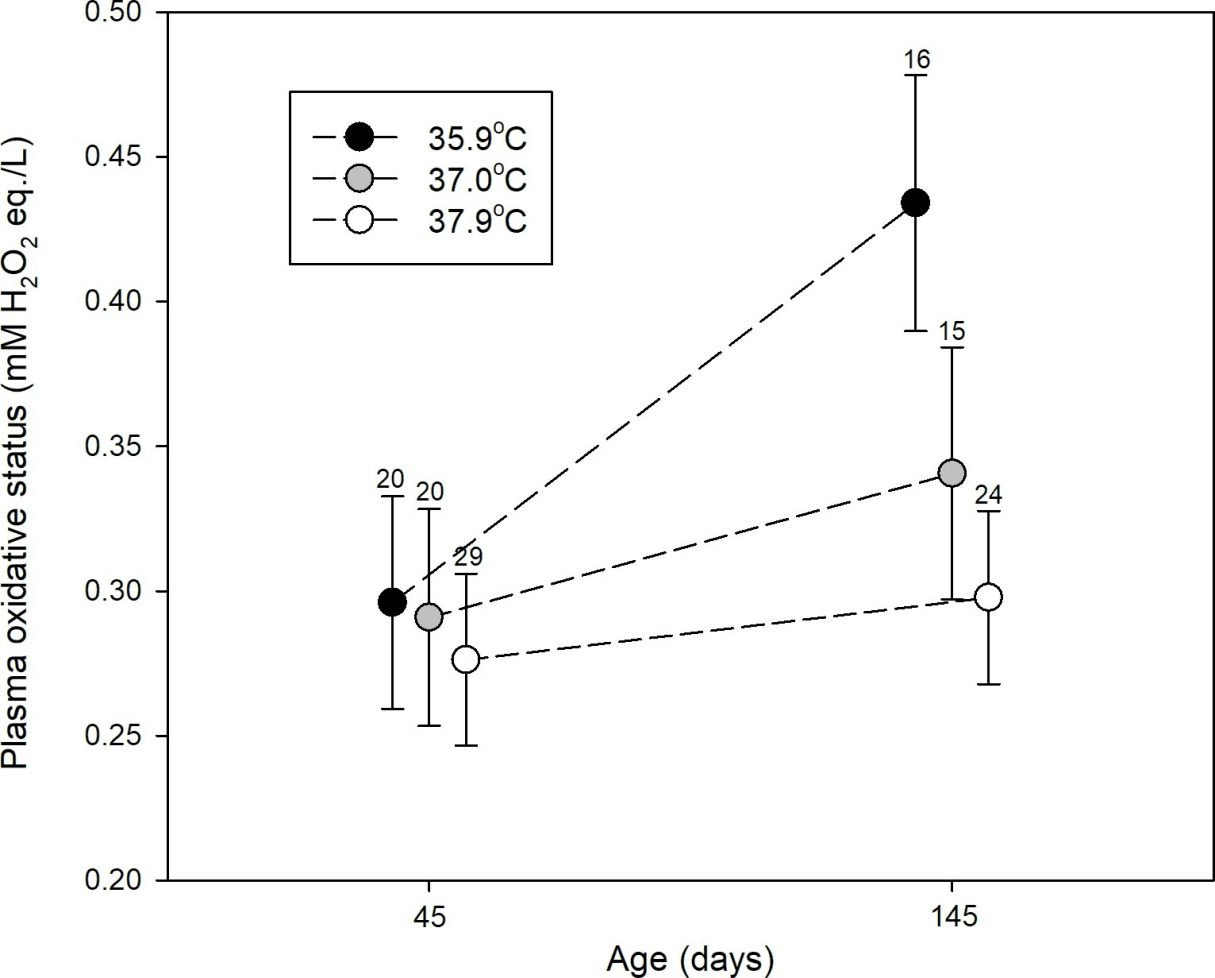
Changes in blood oxidative status are temperature dependent. Plasma concentration of ROMs at day 45 and day 145 for birds from eggs incubated at 35.9°C (black), 37.0°C (grey) and 37.9°C (white). Shown are means ± SE. Numbers above the error bars indicate sample size.

The experimental treatment did not significantly affect the plasma concentration of ROMs at day 45 (P = 0.74), nor was there a significant difference between the sexes (P = 0.20). However, there was an increase in plasma ROMs from day 45 until day 145 and particularly so within the low temperature treatment (Fig 3). Consequently, at day 145, the concentration of plasma ROMs differed significantly between treatment groups (P = 0.05, Table 4). Birds from the 35.9°C treatment group displayed the highest concentration of ROMs (Fig 3), although only significantly higher than birds from the 37.9°C group (post-hoc, P = 0.02, 35.9 vs 37.0°C: P = 0.11, parameter estimates (on log-scale): 35.9°C: -0.387 ± 0.063, 37.0°C: -0.527 ± 0.063, 37.9°C: -0.587 ± 0.052). No difference was found between the sexes (P = 0.96). See Table 4 for full model details. Pairwise t-tests using only individuals with measurements at both ages confirmed that there was a significant increase only in the lowest temperature treatment (35.9°C: t = -2.84, df = 14, p = 0.013, 37.0°C: t = -0.64, df = 14, P = 0.53 and 37.9°C: t = -0.023, df = 23, P = 0.98).

**Table 4:** Results of linear mixed effects models on plasma oxidative status (concentration of ROMs) at 45 and 145 days of age.

	Estimate ± SE	χ^2^	df	p
Day 45				
Treatment	NS	0.08	2	0.96
Sex	NS	1.20	1	0.20
Treatment*sex	NS	0.61	2	0.73
Day 145				
** Treatment**	**-0.387 ± 0.063**	**5.95**	**2**	**0.05**
Sex	NS	0.001	1	0.96
Treatment*sex	NS	4.33	2	0.11

Significance (p) is based on likelihood ratio tests and parameter estimates are given for variables retained in the final model. Estimates are given for Treatment = 35.9°C. Significant term highlighted in bold. See Methods and Results for further details.

## Discussion

### Pre-natal and post-natal growth

Although a lower incubation temperature increased embryonic developmental time (i.e., incubation time), we found that the body mass of hatchling zebra finches did not differ between treatment groups. Because the amount of energy required for physiological maintenance processes increase with incubation time [15, 17, 50] one could expect that a lower temperature may force the embryos to invest more energy into maintaining body functions over a longer period, and hence allocate energy away from tissue growth and maturation. This is indeed what was found by Olson et al. [16], who reported that zebra finch embryos incubated at a mean temperature of 35.4°C exhibited a reduced ability to convert yolk solids into tissue and consequently had a smaller body mass at embryonic day 12, compared to controls (incubated at 37.5°C). However, Olson et al. [16] did not report if this affected the body mass of the hatched chicks. Our study, with the lack of treatment effect on body mass, supports the view that incubation temperature in zebra finches is not affecting the body mass of the chicks. This is further supported by other experimental studies on for example tree swallows (*Tachycineta bicolor*, [22]), blue tits (*Cyanistes caerulens* [20]), wood ducks (*Aix sponsa*, [19]) and Japanese quails (*Coturnix japonica*, [51]). In a study on zebra finches, Wada et al. [52] also found no differences in body mass of hatchlings from eggs that had been incubated at 36.2°C and 37.4°C for the full duration of the incubation period. Hence, our data support the growing body of evidence that egg temperature during incubation has no effect of hatching body mass. Recently, Rubin et al. [53] suggested this to be attributed to a limit to how small an individual’s body mass can be while still surviving the hatching process.

Contrary to our predictions, our experimental treatment was unsuccessful in significantly affecting post-natal growth and thus causing permanent differences in body mass or body size (Table 3, Fig 1). Previous experimental work on e.g. tree swallows and wood ducks have shown that chicks from eggs incubated at lower than normal temperatures hatch with a body mass indistinguishable to that of controls, but show reduced growth patterns of body mass during the nestling period [19, 22]. Such long-term effects on adult body mass have also been reported in Japanese quails, where individuals from eggs incubated at a low temperature (36.0°C) where lighter than control birds (incubated at 37.5°C) at 55 days of age [51]. In contrast, Nord and Nilsson [20] found no effect of incubation temperature (eight days at either 35.0, 36.5 or 38.0°C) on body mass in nestling blue tits (*Cyanistes careuleus*) measured until ~14 days of age. In zebra finches, nestlingfrom eggs incubated at 36.2°C were lighter at 2 and 5 days of age compared to those incubated at 37.4°C [52, 53]. However, this difference did not persist into adulthood (~266 days of age). In contrast, Stier et al. [54] found no effect of incubation temperature on body mass in zebra finches at any age. Our results therefore complement these studies, suggesting that variation in incubation temperature within the natural range does not have a long-lasting negative impact on body mass growth in the zebra finch. A low incubation temperature has also been shown to negatively influence nestling tarsus growth [19, 20, 22]. However, no such effect on tarsus growth was found in our zebra finches. It is, however, noteworthy that although differences in growth parameters (i.e., K, A and I), body mass or tarsus length were not statistically significant, birds from the lowest temperature treatment were on average lighter and smaller in size (i.e., tarsus length) throughout life compared to birds from the highest temperature treatment (Fig 1A and 1B). Particularly, the largest differences in body mass and tarsus length are evident early in the nestling period, at day 10 (Fig 1A and 1B). Nestlings from eggs incubated at 35.9°C also displayed a considerably lower body condition index at day 10 compared to the two other treatment groups (Fig 1C) indicating that these birds exhibit a more rapid growth of tarsus length compared to that of tissues contributing to body mass. However, for both body mass and tarsus length the differences that were evident at day 10 become much smaller with age (Fig 1A and 1B). A low incubation temperature therefore only seems to have had a small effect on the intrinsic capacity for growth in our zebra finches, an effect that could not be detected without a larger sample size.

### Self-maintenance and physiological senescence

We found metabolic rate at day 15 to be highest in chicks that had been incubated at the intermediate temperature (37.0°C, Fig 2). In their study on the blue tit, Nord and Nilsson [22] showed that ~14 day old nestlings from eggs incubated for eight days at a low temperature (35.0°C) had a significantly higher resting metabolic rate compared to those incubated at higher temperatures (36.5 and 38.0°C). Wada et al. [52] similarly found a low incubation temperature (36.2°C) to cause an elevated metabolic rate in 25-day-old female zebra finches. Why birds belonging to our intermediate temperature group displayed the highest metabolic rates is difficult to explain, as we could not detect any effect of the experimental treatment on growth. Nevertheless, the difference in metabolic rate at day 15 did not persist into or throughout adulthood (Fig 2). Similarly, Wada et al. [52] also did not find a long-lasting effect of incubation temperature on metabolic rate (from 25 until 177 days of age). Hence, variation in incubation temperature within the natural range therefore does not seem to be able to permanently alter the metabolic phenotype of offspring in the zebra finch. Contrary to these results, a study on the Japanese quail [51] found long-term effects on BMR in individuals that had been incubated at a low temperature (36.0°C) compared to those incubated at the control temperature (37.5°C). Further studies would be needed to explore this species difference in response.

There are good indications that measuring oxidative damage using blood plasma reflects oxidative damage in other tissues [55], although the generality still has to be proven [31]. Hence, we assume our plasma oxidative measurements are representative of the true oxidative status of the birds. Our experimental treatment did affect plasma oxidative status (Table 4), with a large increase in plasma ROMs from day 45 until day 145 in birds incubated at the lowest temperature (Fig 3) indicating a differential accumulation of oxidative damage with age during an age-interval when most of the dying-off in the low-temperature group occurred [33]. This suggests that the birds are experiencing an accelerated physiological deterioration being mediated through oxidative stress. Resistance to oxidative stress has been shown to decrease with age in the zebra finch [56] and a high resistance to oxidative stress has also been associated with a higher annual survival in barn swallows (*Hirundu rustica*, [57]) and alpine swifts (*Apus melba*, [58]). Hence, a lower investment in e.g. antioxidant defenses or an accelerated rate of decline in antioxidant capacity with age could explain the particularly large increase in plasma ROMs in birds incubated at the lowest temperature, in addition to explaining the effect on survival. However, measures of antioxidant capacity in our birds is needed to confirm such a scenario.

Given that antioxidant defenses and repair mechanisms are inadequate to counteract the oxidative damage to mitochondria, the rate of decline in the intensity of BMR with age could be expected to be proportional to the accumulated damage [34, 59]. However, despite the observed effect on oxidative damage (i.e., plasma concentration of ROMs), the experimental treatment did not influence the rate of metabolic aging in our zebra finches. Contrary to our predictions, the birds do not seem to have experienced any differential loss of mitochondrial function with age due to accumulation of oxidative damage. On the other hand, oxidative stress is managed through a vast array of mechanisms including both intra-cellular and extra-cellular components [27] and multiple biomarkers of oxidative stress may potentially contain unique information about oxidative status [31, 60]. Therefore, although the plasma concentration of ROMs may reflect oxidative damage, we cannot be certain that this reflects damage to mitochondria. Although zebra finches are known to experience metabolic aging [37], there does not seem to be clear relationship between energy metabolism, rates of metabolic aging and survival in this species [38]. The rate of metabolic aging has also been found not be related to individual variation in reproductive senescence in the great tit [61]. Hence, the differences in survival found among birds included in the present study [33] may therefore not be reflected in the rate of metabolic aging, but rather depend on other unmeasured causes of death. The fact that most of the mortality in all three treatments occurred before approximately 150 days of age [33], further strengthen this latter point. A potential cause could be pre-natal telomere shortening [43].

## Conclusions

Because incubation temperature plays a vital part in avian development, deviations from the optimal temperature is likely to be an important source of variation in life-history trajectories of offspring. In the present study we find some indications that a sub-optimal incubation temperature negatively influences body growth and the investment in somatic maintenance. Although incubation temperature did not significantly affect final body mass or size, a low incubation temperature seems to have negatively influenced the capacity to mitigate oxidative damage with advancing age. This apparent reduction of somatic maintenance was not, however, accompanied by an accelerated rate of metabolic aging. Knowing that the birds which had been incubated at the lowest temperature suffered a lower survival these birds clearly paid some cost in terms of physiological deterioration. The present study has shown that this was possibly independent of body growth and basal metabolic tare. Our result highlights the complex nature of the aging process and future studies investigating the effects of incubation temperature on long-term physiological performance therefore need to assess multiple measures of individual state. Future studies should also investigate exactly how and at which life-stage variation in incubation temperature could influence such an investment in somatic maintenance processes.

## Supporting information

S1 Table Number of chicks with the associated number of mothers distributed across treatment groups. (PDF)

S2 Table Whole-body BMR measured at four different ages. (PDF)

S3 Table Biometric measurements of birds belonging to the three experimental treatment groups. (PDF)

S4 Table Blood oxidative status at age 45 and 145. (PDF)
