# Supplementary material for: Incubation temperature and physiological aging in the zebra finch
Source: PLoS One. 2021 Nov 29;16(11):e0260037. doi: 10.1371/journal.pone.0260037 (PMC8629242; doi:10.1371/journal.pone.0260037)
Supplement: S1 Table — (PDF) [file pone.0260037.s001.pdf]

S1 Table. Number of chicks with the associated number of mothers distributed across treatment groups.

Treatment - incubation temperature	Age (days) at measurement			
	0 - 20	45	145	975
One clutch				
35.9ºC	5 (4)	5 (4)	2 (2)	1 (1)
37.0ºC	4 (4)	3 (3)	3 (3)	2 (2)
37.9ºC	10 (5)	10 (5)	8 (5)	4 (4)
Two clutches				
35.9 – 37.0ºC	14 – 15 (6)	10 – 12 (5)	6 – 10 (5)	3 – 7 (5)
37.0 – 37.9ºC	2 – 4 (1)	2 – 4 (1)	3 – 4 (1)	0 – 2 (1)
37.9 – 35.9ºC	9 – 7 (5)	7 – 6 (5)	6 – 1 (5)	4 – 0 (4)
Three clutches				
35.9 – 37.0 – 37.9ºC	9 – 9 – 10 (4)	8 – 9 – 9 (4)	7 – 6 – 9 (4)	3 – 3 – 5 (4)

Females laying multiple clutches (two or three) had each clutch incubated under different experimental temperatures. Numbers in parentheses indicate the number of females.
