# Supplementary material for: Incubation temperature and physiological aging in the zebra finch
Source: PLoS One. 2021 Nov 29;16(11):e0260037. doi: 10.1371/journal.pone.0260037 (PMC8629242; doi:10.1371/journal.pone.0260037)
Supplement: S2 Table — (PDF) [file pone.0260037.s002.pdf]

S2 Table. Whole-body BMR measured at four different ages.

	Incubation temperature		
	35.9°C	37.0°C	37.9°C
Day 15	42.515 ± 0.129 (32)	47.793 ± 1.092 (27)	44.639 ± 1.237 (29)
Day 45	41.094 ± 1.346 (28)	41.813 ± 1.194 (25)	43.289 ± 1.109 (31)
Day 145	41.204 ± 2.107 (16)	40.471 ± 1.044 (19)	41.746 ± 1.430 (26)
Day 975	36.245 ± 1.679 (7)	35.055 ± 1.608 (12)	39.045 ± 1.496 (15)

Values (mL O_2 h^{-1}) are presented as mean ± SE. Sample sizes are shown in parentheses. The obtained measures of whole-body metabolic rate were used to calculate a mass-corrected metabolic rate that was used in the statistical analyses. See Methods for further explanation.
