# Supplementary material for: Incubation temperature and physiological aging in the zebra finch
Source: PLoS One. 2021 Nov 29;16(11):e0260037. doi: 10.1371/journal.pone.0260037 (PMC8629242; doi:10.1371/journal.pone.0260037)
Supplement: S3 Table — (PDF) [file pone.0260037.s003.pdf]

S3 Table. Biometric measurements of birds belonging to the three experimental treatment groups.

		Incubation temperature		
		35.9°C	37.0°C	37.9°C
Day 0	Body mass	0.973 ± 0.029 (35)	0.943 ± 0.031 (30)	0.985 ± 0.028 (33)
Day 10	Body mass	10.12 ± 0.34 (35)	10.51 ± 0.35 (30)	11.20 ± 0.26 (33)
	Tarsus	12.84 ± 0.20 (35)	12.76 ± 0.24 (30)	13.31 ± 0.13 (33)
Day 20	Body mass	12.71 ± 0.25 (35)	12.80 ± 0.29 (30)	13.37 ± 0.33 (33)
	Tarsus	14.56 ± 0.13 (35)	14.72 ± 0.12 (30)	14.90 ± 0.14(33)
Day 45	Body mass	15.42 ± 0.40 (29)	15.22 ± 0.43 (26)	16.45 ± 0.38 (30)
	Tarsus	14.72 ± 0.12 (29)	14.80 ± 0.15 (26)	15.04 ± 0.13 (30)
Day 145	Body mass	15.81 ± 0.72 (16)	16.00 ± 0.50 (19)	16.34 ± 0.48 (26)
	Tarsus	14.64 ± 0.18 (16)	14.80 ± 0.19 (19)	14.87 ± 0.13 (26)
Day 975	Body mass	15.74 ± 0.59 (7)	16.44 ± 0.92 (12)	17.79 ± 0.59 (15)
	Tarsus	14.45 ± 0.21 (7)	14.91 ± 0.23 (12)	15.13 ± 0.16 (15)

Measurements (mean ± SE) were made at six different ages ranging from hatching (day 0) until 975 days of age. Body mass is expressed in grams and tarsus length is expressed in millimeters. Number in parentheses indicates sample size.
