# Supplementary material for: Incubation temperature and physiological aging in the zebra finch
Source: PLoS One. 2021 Nov 29;16(11):e0260037. doi: 10.1371/journal.pone.0260037 (PMC8629242; doi:10.1371/journal.pone.0260037)
Supplement: S4 Table — (PDF) [file pone.0260037.s004.pdf]

**S4 Table. Blood oxidative status at age 45 and 145.**

| | Treatment / incubation temperature | | |
|---|---|---|---|
| | 35.9ºC | 37.0ºC | 37.9ºC |
| Day 45 | 0.296 ± 0.037 (20) | 0.291 ± 0.037 (20) | 0.276 ± 0.030 (29) |
| Day 145 | 0.434 ± 0.044 (16) | 0.341 ± 0.044 (15) | 0.298 ± 0.030 (24) |

Shown are concentration (in mmol $L^{-1}$ $H_2O_2$) of hydroperoxides (ROMs) in plasma of zebra finches taken at the age of 45 and 145 days of age. Values are presented as mean ± SE. Sample sizes are shown in parentheses.
